# Supplementary material for: Study on the Densification of Osmium by Experiment and First Principle Calculations
Source: Materials (Basel). 2022 Nov 13;15(22):8011. doi: 10.3390/ma15228011 (PMC9697943; doi:10.3390/ma15228011)
Supplement: Supplementary file 1 [file materials-15-08011-s001.zip › materials-1972983-supplementary.pdf]

Supplementary Material for

Study on the Densification of Osmium by Experiment and First Principle Calculations

Yunfei Yang, Junhao Sun, Wei Liu, Peng Hu, Ruimin Zhang, Hexiong Liu, Junyan Gao, Jinshu Wang*

Faculty of Materials and Manufacturing, Beijing University of Technology, Beijing, 100124, China.

*Corresponding author: wangjsh@bjut.edu.cn

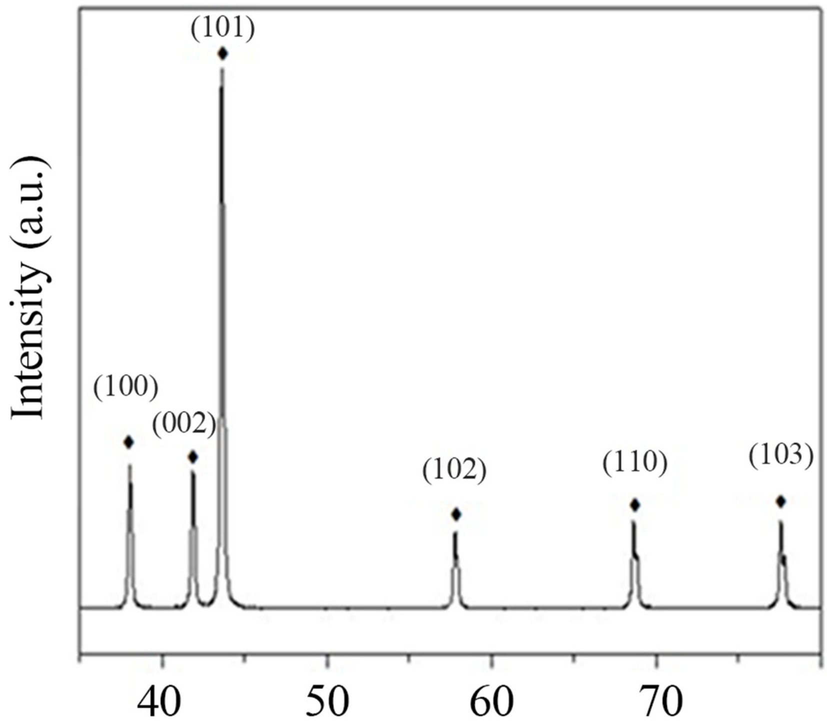

Figure S1. The XRD of purified Osmium raw powders.

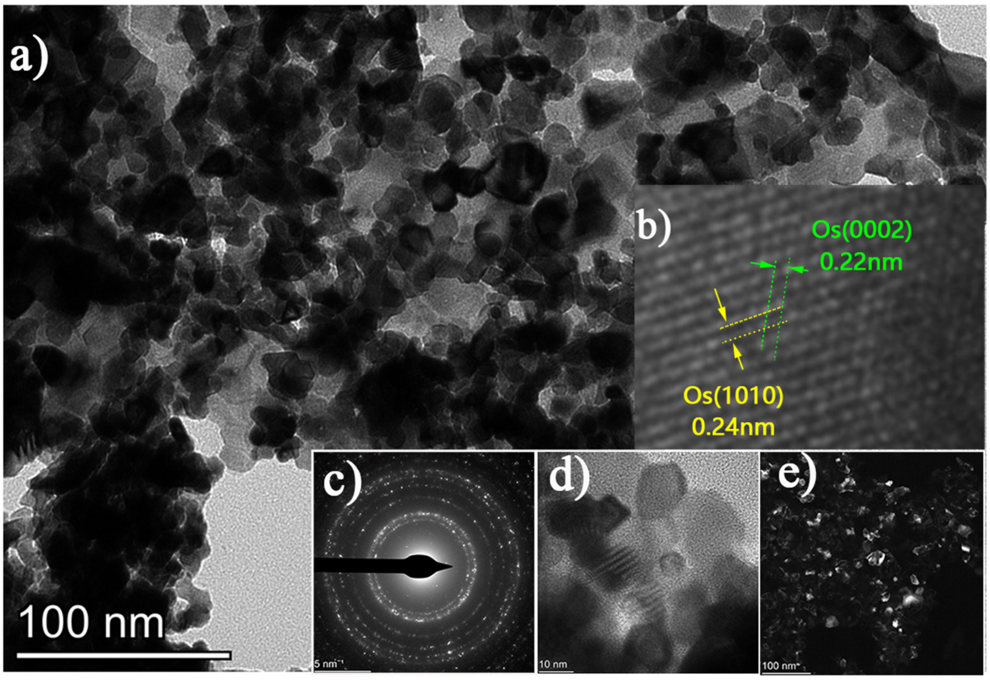

Figure S2. (a) TEM, (b) HRTEM, (c) SAED, (d) BF TEM and (e) DF TEM of purified Osmium raw powders.

Figures S1 and S2 show the XRD and TEM analysis results of the source material, respectively. It can be seen that the main component of the powder is metallic osmium, and no osmium oxide appears. At the same time, the powder is agglomerated but the grain size is very small and is still nano-scale.
